# NCR-PCOPGene: An Exploratory Tool for Analysis of Sample-Classes Effect on Gene-Expression Relationships

**DOI:** 10.1155/2008/789026

**Published:** 2008-12-10

**Authors:** Juan Cedano, Mario Huerta, Enrique Querol

**Affiliations:** Departament de Bioquímica i Biología Molecular, Institut de Biotecnologia i Biomedicina, Universitat Autònoma de Barcelona, 08193 Bellaterra, Barcelona, Spain

## Abstract

*Background*. Microarray technology is so expensive and powerful that it is essential to extract maximum value from microarray data. Our tools allow researchers to test and formulate from a hypothesis to entire models. *Results*. The objective of the NCRPCOPGene is to study the relationships among gene expressions under different conditions, to classify these conditions, and to study their effect on the different relationships. The web application makes it easier to define the sample classes, grouping the microarray experiments either by using (a) biological, statistical, or any other previous knowledge or (b) their effect on the expression relationship maintained among specific genes of interest. By means of the type (a) class definition, the researcher can add biological information to the gene-expression relationships. The type (b) class definition allows for linking genes correlated neither linearly nor nonlinearly. *Conclusions*. The PCOPGene tools are especially suitable for microarrays with large sample series. This application helps to identify cellular states and the genes involved in it in a flexible way. The application takes advantage of the ability of our system to relate gene expressions; even when these relationships are noncontinuous and cannot be found using linear or nonlinear analytical methods.

## 1. Introduction

DNA microarray technology enables high-throughput gene-expression analysis, which allows researchers to compare the activity of genes in multiple cellular conditions. There are several relevant web applications for microarray analysis, that is, GEO [1], BIOREL [2], ArrayExpress [3], and MicroGen [4]. Currently, most tools try to extract biological information from such high-throughput expression data combining information from coexpressed genes [5] as well as additional annotations extracted from Gene-Ontology [6], phylogenetic information [7], or pathway data [8]. In this paper, a different and complementary new approach based on the effect of the experiments on the fluctuations of gene-expression relationships is proposed.

The suitable data for our type of analysis can be provided by **(a)** temporal series, useful to study synchronous cellular events, and **(b)** serial analysis of gene-expression samples under different conditions (i.e., chemotherapy, temperature, radiation, starvation, etc.) which are more useful for studying asynchronous events. The progressive increase of microarray sample series [9] motivates a more thorough analysis of expression relationships and gene dependencies throughout these large series, trying to rescue global gene behaviours, cellular states and phenotypes. The GEO database [1] facilitates the study of the microarray experiments grouped into predefined classes introduced by the microarray authors. Nevertheless, if the researcher wants to understand the microarray experiments effect on the expression relationships and elucidate hidden cell states, he/she needs a more specific approach. To study the effect of the microarray experiments on the relationships among different sets of genes, our web tool allows the user to define flexible sample classes. It involves a significant new approach by going from the linear, nonlinear, and mutually exclusive gene relationships to the complex, noncontinuous dependence among genes or sets of genes.

Our strategy begins from the analysis of the “continuous gene-expression relationships” (abbreviated in the paper as “gene relationships” or “expression relationships”). This analysis provides the “inner pattern” of the expression relationships. This “inner pattern” describes the relationship in an *n*-space (sample space of *n* dimensions), where each axis represents the expression level of each one of the genes which are being related, and from a data cloud with the microarray experiments. This pattern analysis detects the lineal, nonlineal, and mutually excluding relationships, providing a series of points in the *n*-space which describe the “inner pattern” of the expression relationship. Beyond this first analysis remains the second objective: the study of the “noncontinuous dependence” among gene expressions. To perform this, the sample classes are defined allowing for the study of their effect on gene expressions and the stated continuous dependencies. This task facilitates relating genes which do not have continuous relationships but rather have local, unidirectional, or other kinds of complex dependence in their expression. The final objective is that the sample classes defined help to identify the hidden cellular states and phenotype changes (and provide the genes involved).

## 2. Methods

Pattern extraction methodologies are very powerful techniques to extract biological knowledge from data, as was shown in our previous work [10], where this approach was used for analysis of populations, diagnosis, and prognosis. In the present work, a useful extension to high-throughput microarray analysis is presented.

### 2.1. Preprocess

When microarray data are uploaded, the inner pattern of the expression relationship of all pairs of microarray genes is calculated automatically. These calculations are made by the principal curves of oriented points (PCOPs) calculus [11] and recognise the linear, nonlinear and mutually-excluding relationships between genes (both genes cannot be overexpressed or underexpressed at the same time, or one gene can be overexpressed if the other one is underexpressed) [12]. The noncorrelation factor between each pair of gene expressions is calculated in the preprocess pattern analysis, leading to the identification of those genes which are more correlated with each gene. How it will lead to the “analysis expansion” around the researcher's genes of interest is shown in Results, and a brief description of the PCOP calculation [11] is given in Section 2.4.

### 2.2. Defining Sample Classes

The researcher could test his/her hypotheses and intuitions by defining the sample classes. The sample-classes definition can be made in the following three different ways.

- Selecting the samples from a range of gene-expression data.
- Clustering the samples from a gene-expression relationship: once the inner pattern among a set of correlated genes of interest is calculated (once again by PCOP calculus [11]), the samples that constitute the different “local behaviours” of this inner pattern can be clustered (and a high correlation implies a better-fitted pattern). Then, the effect of these conditions can be studied on any other genes relationship, where these genes could not be correlated with the set initially used to cluster the microarray experiments. Note that although in the preprocess PCOP is calculated for two dimensions only (a pair of genes), now it is calculated for *n* ≥ 2 dimensions (the *n* genes of the gene set).
- Classifying the microarray experiments into different classes using previous knowledge.

### 2.3. Colouring the Sample Classes in a Genes Relationship

**Figure 1:**
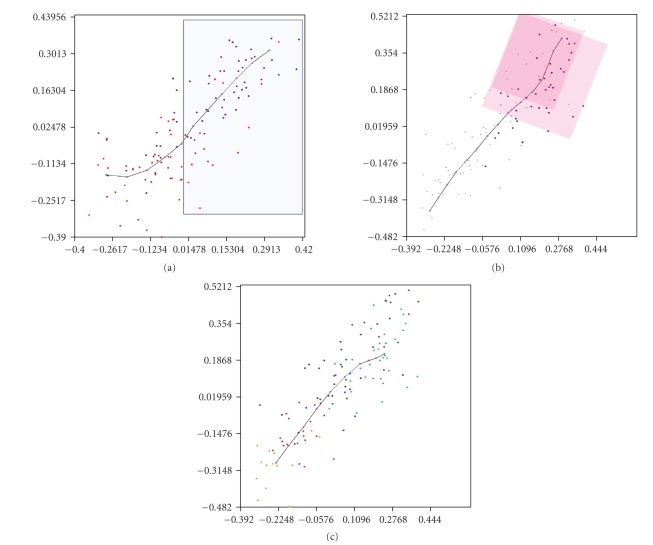
Sample class definitions using the PCOPGene web interface. *Example 1*: in the *SGC(GUCY1B3)* and *Q6(QSN6)* relationship ((a) f* = 0.08), the samples of the two extremes of the *SGC/Q6* inner pattern are clustered into two classes by clicking on the POPs, these classes are coloured in the *GATA3* and *AML1(RUNX1)* relationship ((b) *f* = 0.10). *Example 2*: in the *GATA3* and *AML1(RUNX1)* comparison window (b), the samples of *GATA3 * over-expressed are grouped with the range-selection facility and coloured in the *SGC* and *Q6* relationship (a). *Example 3:* in the *GATA3*, *AML1(RUNX1)*, *SGC* and *Q6* relationship ((c) *f* = 0.28), the plot of the *SGC* and *Q6* plane is shown. The coloured classes shown have been obtained by clustering the microarray experiments according to their correlation. These classes represent differentiated cell processes with different genes involved. (f* = non-correlation factor provided by the PCOP calculus [10]).

Now, the graphical interface facilitates the visualisation of the defined-classes effect on gene relationships by colouring the samples (as is shown in Figure 1) for every set of genes of interest with the intention of studying their effect. The study of this effect is especially relevant in the nonlinear relationships in order to understand the biological sense of the slope change, possibly due to a phenotype change.

The class definitions of types (i) (selecting the samples from a range of gene-expression values) and (ii) (clustering the samples from a gene-expression relationship) are made using these graphical visualisations of the initial-genes-of-interest pattern analysis. Type (iii) is made using the web interface that enquires for the samples belonging to each class, or directly uploading the file.

### 2.4. Defining the Sample Classes from a Nonlinear Expression Relationship

The system makes use of the principal curves of oriented points (PCOPs) calculus to obtain the nonlinear inner pattern [11, 13] used to cluster the samples (remember that PCOP calculus is also used to obtain the correlation degree between all pairs of microarray genes). The analysed variables with the PCOP method can be independent because the method uses a hidden variable for ordering the data (in contrast to other nonlinear analyses like regression curves) which is suitable for gene-expression comparisons [14]. PCOP is defined by the generalisation, at the local level, of the principal-components variance properties. From the sample-space data of *n* dimensions (one per gene expression), our system obtains discretised states named “principal oriented points” (POPs), which represent this principal component generalisation at the local level or local area. The series of principal oriented points (POPs) obtained makes up the principal curve of oriented points (PCOPs) or inner pattern of the data cloud [11, 13]. The final series of POPs obtained will minimise the dispersion degree of the samples around PCOP [11, 13]. As a result, even with no isotropic distribution of the data, we obtain a very realistic inner pattern, compared, for example, with methods like least squares, and a very accurate data-dispersion measurement (the noncorrelation factor), compared with artificial intelligence methods [11, 15].

Next, when the user selects the POPs (the different discretised states obtained in the PCOP calculus) using the graphical interface, the samples belonging to each POP scope or local area are selected. One of the main keys of PCOP calculus is precisely the scope detection of the local area of each POP, as has been explained in a previous work [11]. In this way, the different sample classes are finally defined based on the “local behaviours” of the gene-expression relationship. For more details about this clustering method based on the fluctuations of the inner pattern, consult our previous work [10].

### 2.5. Gene Search Based on the Distribution of the Classes along the Gene-Expression Range

To make it easier to relate the genes in a noncontinuous approach, the user can carry out a gene search based on the expression level required for each class defined. This noncontinuous approach leads to the correlation of gene expressions that cannot be related by the continuous analysis facilitated by the pattern analysis (as shown in the actual examples described in Section 3). In the “class-distribution” search, the genes can be searched for some of the defined classes by being upregulated or downregulated, with respect to the basal value, or by being disjointed, overexpressed, or underexpressed, with respect to some of the other classes. All possible combinations are allowed where different combinations will supply different gene sets.

### 2.6. Contextualisation Consulting the GEO Database

In order to get complementary information from other public microarray data, the GEO database could be consulted. These queries attempt to know if the genes supplied by the “class-distribution” search tool (genes that follow the user-defined class distribution in their expression) are gene markers in the GEO datasets. To achieve this information, the NCR-PCOPGene queries the microarray “gene-centric” GEO Profiles [1]. The profiles across all GEO datasets where the query gene displays significant expression differences among the GEO-predefined sample classes are obtained. In this way, the queried gene can be considered, for example, as a marker of osmotic stress in a microarray to analyse cellular stress response, or as a marker of metastasis in another microarray to analyse disease states, and so forth. These query-gene properties can then be assigned to the sample classes obtained with the NCR-PCOPGene.

### 2.7. Availability and Requirements

1. *Project name:* PCOPGene.
2. *Project home page:* http://ibb.uab.es/revresearch/
3. * Operating system(s):* Web-based application.
4. *Programming language:* PHP, Java, flash script, CGI, C++, Matlab (interfaces for Matlab users).
5. *Requirements:* Mozilla 5.0, sea monkey 1.0, Firefox 1.0 or Explorer 6.0. flash plug-in 7.0 and Java 1.3.1 or higher versions.
6. * Licence:* free access; source codes available in the web.
7. *Any restrictions to use by nonacademics:* we prefer no use for profit.

A demo user for visualising the above examples is available. A multimedia tutorial is provided to describe the application use and it is indexed from the application help icon. For mathematical and computational details, technical reports are also available in the web.

## 3. Results

Let us describe some real-use cases of the NCR-PCOPGene and the relevance of the new knowledge supplied (the gene-expression dependence put forward in the examples are new and unknown, until now). The three ways to define the sample classes and a basic-analysis procedure have been used.

### 3.1. Microarray Data

The suitable data to be analysed by the NCR-PCOPGene are microarrays with large synchronous or asynchronous sample series. The analysis presented in the paper uses microarray data provided by the National Cancer Institute (USA) [16]. They correspond to the profiles of 9703 cDNAS representing ~8000 unique genes of 60 cell-lines, in relation to the activity profiles of 1400 drugs. They provide a resulting table of 1376 genes and 118 compounds with the most representative substances and genes normalised for the 60 cell-lines (suitable data for knowledge discovery using our tools).

### 3.2. Basic Analysis Procedure

The analysis begins in the researcher's genes of interest, usually gene markers of a specific disease, cell state, or function. As was commented on in Section 2, the correlation factors between all microarray pairs of genes (for the linearly, nonlinearly, and mutually excluding relationships) are automatically calculated when the microarray data are uploaded (the preprocess). Thus, for each gene the system provides its rank of best-correlated genes and the user can expand the initial correlated-gene-set based on these ranks [14]. And then finally, the user can launch the PCOP calculus of the gene set.

Once the pattern analysis of the query genes has been performed, the graphical interface will show their expression-relationship, inner pattern, and their fluctuations. Also, their noncorrelation factor of the set is provided (remember that a better correlation among the initial gene-set implies an inner pattern that fits better, facilitating the detection of the fluctuations and slope changes, and also the clustering of the different microarray experiments associated with each one).

To find the expression dependence of these initial sets of correlated genes with other genes either linearly or nonlinearly correlated with the first ones (on the contrary, it is easier and faster to perform PCOP analysis directly), the user should proceed as follows: first, he/she must discretise the continuous relationship among the initial gene set forming the different classes (by simply clicking on the POPs along the relationship's inner-pattern in the plot interface); second, applying the “classes-distribution” search tool for a certain distribution of that discretisation, the genes that follow the required distribution in their expressions are obtained. Finally, the researcher can now perform the pattern analysis of the genes provided and observe, in its interface visualisation, the effect of each sample class on its expression relationship (with the samples of the classes coloured, as in Figure 1). This procedure will show the noncontinuous dependence among the genes provided by the search and the initial gene set in the manner specified in the search. If the distribution required in the search is changed, the genes provided and their noncontinuous dependence, with respect to the initial ones, will vary too.

### 3.3. Example 1: Defining the Sample Classes from a Nonlinear Genes Relationship

Let us now look at a real analysis. We wish to relate the Soluble Guanylate Cyclase Beta1 3 (*SGC*) and Quiescin *Q6 (Q6)* genes (our genes of interest). The *SGC* is underexpressed in cellular stress [17], whereas *Q6* is overexpressed in the last phases of tissue remodelling [18]. Additionally, we would like to relate this pair of genes to GATA-binding protein 3 (*GATA3*) and acute myeloid leukaemia 1 (*AML1*, *RUNX1*). *GATA3* is involved in growth control and the maintenance of the differentiated state in epithelial cells [19]. The impairment of the *AML1* function deregulates the pathways leading to cellular proliferation and differentiation [20]. The two gene sets show a correlation between their respective members (noncorrelation factors of *SGC* versus *Q6* = 0.08; *GATA3* versus *AML1* = 0.1) and their inner pattern can be calculated and visualised in the graphical interfaces, as is shown in Figure 1. The problem is that these two sets of genes are neither linearly nor nonlinearly correlated (noncorrelation factor for *SGC* versus *Q6* versus *GATA3* versus *AML1* = 0.28). But perhaps they are maintaining a noncontinuous dependence in their expressions, and we cannot discern it with the analysis for continuous data-clouds. However, we can try to find it using the classes' definition.

To perform this, two clusters are built from the *SGC* and *Q6* relationship by selecting the POPs located in the two extremes of their inner-pattern relationship, one corresponding to the cellular-stress samples and the other to the tissue-remodelling samples (Figure 1, *SGC* and *Q6* relationship). These clusters will constitute two different sample classes.

**Figure 2:**
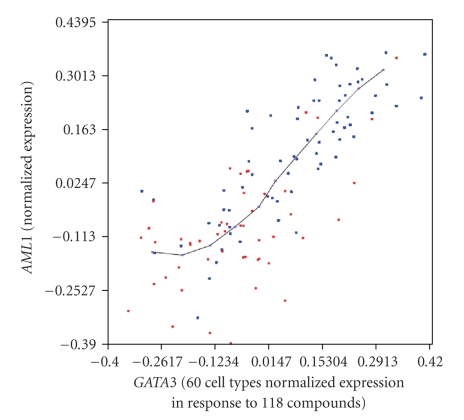
*Example 1:* effect of classes derived from *Q6* and *SGC* on *GATA3* and *AML1* expression. In the picture is shown the *GATA3* (*x* axis) and *AML1* (*y* axis) relationship, where the data-cloud shows the microarray experiments and the PCOP, connecting the POPs, describes the inner pattern of the expression relationship. The samples under-expressed in the *Q6* and *SGC* relationship are coloured in red, and the samples over-expressed in the *Q6* and *SGC* relationship are coloured in blue. This points out that the under-expression of *Q6* and *SGC* implies an under-expression of *GATA3* and *AML1*, but that an over-expression of *Q6 * and *SGC* does not always imply an over-expression of *GATA3* and *AML1*. The data set represents the activity of 118 substances normalised for 60 cell-lines [16].

Now, the classes are applied to the *GATA3* and *AML1* relationship, painting their respective samples with red and blue colours (Figure 2). As can be observed in Figure 2, almost all samples corresponding to cellular stress (red) appear with an underexpression of *GATA3* and *AML1*, indicating that the two genes are not overexpressed in cellular stress. However, the tissue-remodelling-class samples (blue) appear along the *GATA3* and *AML1* relationship as being over- and underexpressed and indicate that some of these tissue-remodelling conditions are affected by the *GATA3* and *AML1* differentiation ways while some are not. This points out that the overexpression of *GATA3* and *AML1* implies an overexpression of *SGC* and *Q6*, but not the opposite. Note that this “unidirectional” relationship is impossible to detect by pattern- or correlation-analysis methods.

### 3.4. Example 2: Defining the Sample Classes from Selecting Expression Ranges

As seen above, there are some experiments involved in tissue remodelling, but not in the *GATA3* differentiation processes. It would be interesting to study them. For this purpose, those samples where *GATA3* is overexpressed were selected (using the graphical interface) to define a new sample class (Figure 1, *GATA3* and *AML1* relationship).

**Figure 3:**
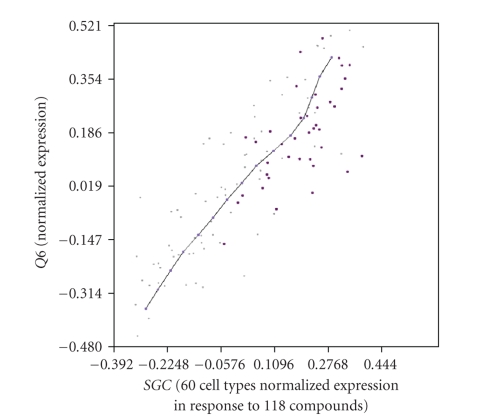
*Example 2:* effect of over-expressed class from *GATA3* and *AML1* on *SGC* and *Q6* expression: *SGC* (*x* axis) and *Q6* (*y* axis) relationship, where the data-cloud shows the microarray experiments and the PCOP, connecting the POPs, describes the inner pattern of the expression relationship. The samples of the class representing the over-expression of *GATA3* and *AML1* are coloured. In this way, the four gene expressions are related, *SGC* and *Q6* linearly, with *GATA3* and *AML1* maintaining a complex noncontinuous relationship with the other two. The data set represents the activity of 118 substances normalised for 60 cell-lines [16].

This class of samples is coloured in the *SGC* and *Q6* relationship, as shown in Figure 3. As we can see, the differentiation induced by *GATA3* is independent of the tissue-remodelling level achieved by the *SGC* and *Q6* relationship. Note that the sample selection from POP is the appropriate method to classify cellular states because the cause or effect of a cell state is the combined expression of multiple genes.

### 3.5. Example 3: Defining the Sample Classes by Classifying the Experiments Using Previous Knowledge

Previous knowledge can arise basically from two different origins: a biological/clinical origin or a statistical one. In our case, the microarray experiments are grouped by their linear correlation using principal components, but other methods like biclustering [21, 22] or Locally Linear Embedding [23, 24] can be used with better accuracy to define the classes. Note that in order to define the classes, the microarray experiments play the variable role and the genes make up the data cloud. Defining the classes by the microarray-experiments correlation, sets of genes are similarly expressed under the conditions of certain experiments (a class), but not under the conditions of the others. Thus, we can establish the hypothesis that each sample class represents a differentiated cell process, with the genes implicated in it acting jointly.

The effect of the defined classes on relationships among genes of interest can now be observed. Colouring these classes in the relationship among the four genes of the above examples (Figure 1, *GATA3, RUNX, SGC,* and *Q6* relationship), and remembering the observations of the above examples, the genes that may act jointly would be (i) the genes involved in cell stress (yellow and red) and (ii) the genes involved in tissue remodelling (green and blue), this last set being divided into genes related to *GATA3* and *AML1* differentiation (green), and those not implicated in it (blue).

### 3.6. Example 4: Gene Search Based on the Distribution of the Classes along Their Expression Range

With the classes obtained in the last example (Figure 1, *GATA3, RUNX, SGC,* and *Q6* relationship) and using the “class-distribution” gene-search tool, it is interesting to search the genes which mark the “transition” from the cell process involved in tissue remodelling without *GATA3* differentiation (blue) to the cell process linked to tissue remodelling with *GATA3* differentiation (green). For this purpose, the following “class-distribution” gene search must be performed: the blue and green classes are overexpressed, with respect to the basal value; the rest of the classes are underexpressed, with respect to the basal value; and the green-class samples are more overexpressed than the blue-class samples.

Furthermore, we can identify gene markers of specific cell processes or pathologies (in relation to their expression levels) from the supplied genes by means of the queries made by the application against the “gene-centric” GEO profiles. Thus, the analysis can be focused on the relevant genes for biomedical user interest.

## 4. Conclusions

NCR-PCOPGene strength resides in **(a)** the flexibility of the classes' definition, due to the nonlinear pattern analysis of gene expressions and the sample clustering along the inner pattern, combined with **(b)** the high-throughput approach of microarray technology, which, by means of the “class-distribution” gene search and the gene-correlation table, leads the researcher to expand his/her analysis. As a result, our application can help to relate gene expressions when their relationships are noncontinuous and cannot be found using linear or nonlinear analytical methods.

The flexibility of the tool leads to the combination of the three ways for determining the classes shown in this paper, to define and redefine the classes. For example, using the “*class definition from a nonlinear genes relationship*” way, the classes can be clustered from two different, uncorrelated, initial gene sets (as long as no sample appears in more than one class). Therefore, the user could search the genes that are partially related to both sets of genes in a specific manner (e.g., being correlated with one set in the underexpression, but with the other in the overexpression analyses). Or initially using the “*previous-knowledge*” way, the user redefines the predefined classes by their effect on the gene-expression-relationships (performing subclasses from the original ones to study subprocesses in which the genes of interest are involved).

In summary, it is a powerful tool to study genes of interest and test researchers' hypotheses by taking advantage of the high-throughput capability of microarray technology.

## References

[1] Barrett T, Suzek TO, Troup DB (2005). NCBI GEO: mining millions of expression profiles—database and tools. *Nucleic Acids Research*.

[2] Antonov AV, Tetko IV, Mewes HW (2006). A systematic approach to infer biological relevance and biases of gene network structures. *Nucleic Acids Research*.

[3] Parkinson H, Sarkans U, Shojatalab M (2005). ArrayExpress—a public repository for microarray gene expression data at the EBI. *Nucleic Acids Research*.

[4] Burgarella S, Cattaneo D, Pinciroli F, Masseroli M (2005). MicroGen: a MIAME compliant web system for microarray experiment information and workflow management. *BMC Bioinformatics*.

[5] Frickey T, Weiller G (2007). Analyzing microarray data using CLANS. *Bioinformatics*.

[6] Nam D, Kim S-B, Kim S-K, Yang S, Kim S-Y, Chu I-S (2006). ADGO: analysis of differentially expressed gene sets using composite GO annotation. *Bioinformatics*.

[7] Frickey T, Lupas A (2004). CLANS: a Java application for visualizing protein families based on pairwise similarity. *Bioinformatics*.

[8] Thimm O, Bläsing O, Gibon Y (2004). MAPMAN: a user-driven tool to display genomics data sets onto diagrams of metabolic pathways and other biological processes. *The Plant Journal*.

[9] Hall PA, Todd CB, Hyland PL (2005). The septin-binding protein anillin is overexpressed in diverse human tumors. *Clinical Cancer Research*.

[10] Cedano J, Huerta M, Estrada I (2007). A web server for automatic analysis and extraction of relevant biological knowledge. *Computers in Biology and Medicine*.

[11] Delicado P, Huerta M (2003). Principal curves of oriented points: theoretical and computational improvements. *Computational Statistics*.

[12] Huerta M, Cedano J, Querol E (2008). Analysis of nonlinear relations between expression profiles by the principal curves of oriented-points approach. *Journal of Bioinformatics and Computational Biology*.

[13] Delicado P (2001). Another look at principal curves and surfaces. *Journal of Multivariate Analysis*.

[14] Huerta M, Cedano J, Querol E (2008). Analysis of nonlinear relations between expression profiles by the principal curves of oriented-points approach. *Journal of Bioinformatics and Computational Biology*.

[15] Sandilya S, Kulkarni SR (2002). Principal curves with bounded turn. *IEEE Transactions on Information Theory*.

[16] Scherf U, Ross DT, Waltham M (2000). A gene expression database for the molecular pharmacology of cancer. *Nature Genetics*.

[17] Roelofs J, Van Haastert PJM (2002). Characterization of two unusual guanylyl cyclases from *Dictyostelium*. *The Journal of Biological Chemistry*.

[18] Thorpe C, Hoober KL, Raje S (2002). Sulfhydryl oxidases: emerging catalysts of protein disulfide bond formation in eukaryotes. *Archives of Biochemistry and Biophysics*.

[19] Usary J, Llaca V, Karaca G (2004). Mutation of *GATA3* in human breast tumors. *Oncogene*.

[20] Michaud J, Scott HS, Escher R (2003). AML1 interconnected pathways of leukemogenesis. *Cancer Investigation*.

[21] Getz G, Domany E (2003). Coupled two-way clustering server. *Bioinformatics*.

[22] Tanay A, Sharan R, Shamir R (2002). Discovering statistically significant biclusters in gene expression data. *Bioinformatics*.

[23] Roweis ST, Saul LK (2000). Nonlinear dimensionality reduction by locally linear embedding. *Science*.

[24] Chao S, Lihui C Feature dimension reduction for microarray data analysis using locally linear embedding.

